# Incidental Papillary Thyroid Microcarcinoma in an Endemic Goiter Area

**DOI:** 10.1155/2016/1784397

**Published:** 2016-02-02

**Authors:** Emin Gürleyik, Gunay Gurleyik, Banu Karapolat, Ufuk Onsal

**Affiliations:** ^1^Department of Surgery, Duzce University Medical Faculty, 81650 Duzce, Turkey; ^2^Haydarpasa Numune Hospital, Istanbul, Turkey

## Abstract

Clinical and pathological characteristics of incidental papillary thyroid microcancer cases, surgical, medical, and nuclear treatment methods, and patients' outcome were studied during follow-up period of 102 months. We studied 37 patients with incidental papillary thyroid microcancer (I-PTM). The surgical procedure was total thyroidectomy in 29 and hemithyroidectomy in 8 patients. Size, multifocality, and bilateralism of PTM foci, thyroid capsule invasion, and presence of lymphovascular invasion were histopathological parameters. We analysed adjuvant medical and nuclear treatment and patients' outcome during follow-up period of 102 (61–144) months. The prevalence rates of I-PTM were 9.4% in 395 thyroidectomy cases. Histopathological examination reported unifocal disease in 30 and multifocal disease in 7 (18%) patients. Multifocal disease was bilateral in 6 (20.1%) patients. The mean size of the PTM foci was 4.88 mm. The rate of thyroid capsule invasion was 5.4%. All patients received a suppressive dose of LT4 to achieve a low serum TSH level. Adjuvant surgical and nuclear treatment was not performed in our cases. We did not find any negative changes in blood chemistry and ultrasound imaging, and any unfavourable events as locoregional and systemic recurrence. In conclusion, diagnosis of I-PTM is common that multifocality and bilateralism appear as pathologic features. The prognosis is excellent after surgical treatment and TSH suppression. Routine adjuvant nuclear treatment is unnecessary in majority of patients.

## 1. Introduction

The incidence of well-differentiated thyroid carcinoma, particularly papillary cancer, has been increasing since the last 20–30 years. The Surveillance, Epidemiology and End Results (SEER) database shows more than a 2-fold increase in thyroid cancer since 1995 [1]. An important contributing factor for the increased incidence of such well-differentiated cancers is the increasing diagnostic rates of papillary thyroid microcarcinoma (PTM). Other factors such as iodination programmes in low iodine intake areas, detailed histopathological examination of the excised thyroid tissue, and the increase in bilateral total excision of the thyroid gland during thyroid surgery have also been attributed to the increasing rates of large (>10 mm) and micropapillary carcinoma [2–5]. A vast majority of PTM cases are incidentally determined on postoperative histopathological examination of the excised thyroid tissue for the surgical treatment of benign thyroid disorders.

PTM is defined as a tumour focus that is ≤10 mm in size. Incidental PTM (I-PTM) is a tumour focus that is clinically unsuspected before thyroid surgery and is identified in the final pathological examination of a thyroidectomy specimen. Therefore, several controversies regarding the need for completion surgery for excision of the remaining thyroid tissue and lymph nodes exist.

The objectives of this study were to describe the incidence and clinical/pathological characteristics of PTM and discuss our experience with I-PTM cases in an endemic goitre area.

## 2. Materials and Methods

### 2.1. Patients and Thyroid Surgery

Surgically treated patients between September 2003 and September 2010 were retrospectively analysed. The study involved 395 surgical patients with benign disease of the thyroid, without any diagnosis of preoperative malignancy. Total thyroidectomy or hemithyroidectomy was performed for the treatment of benign thyroid diseases. A total of 37 patients with PTM incidentally diagnosed on postoperative histopathological examination of the excised thyroid tissue were analysed for assessing the rate of incidental diagnosis of PTM and their demographic features and the surgical procedures used for their treatment.

### 2.2. Histopathology

Histopathological parameters were established by microscopic examination, including the size of PTM, location in the thyroid gland, multifocality and bilateralism in the thyroid lobes, thyroid capsule invasion, presence of lymphovascular invasion (LVI), lymph node metastasis, and tumour recurrence.

### 2.3. Adjuvant Treatment

As an adjuvant treatment, we analysed completion thyroidectomy for I-PTM cases with unilateral hemithyroidectomy, l-thyroxin (LT4) treatment for the suppression of thyroid stimulating hormone (TSH), and radioiodine (RAI) treatment.

### 2.4. Follow-Up

Patients with I-PTM were followed up for 12 years, mean 102 (61–144) months.

#### 2.4.1. First and Third Month Postoperatively

Biochemical analyses for serum TSH and free thyroxin (FT4) were performed in order to determine the suppressive dose of LT4 (suppression of TSH at a level of <0.25 uIU/mL).

#### 2.4.2. Sixth Month Postoperatively

Biochemical analyses for serum TSH, FT4, thyroglobulin (Tg), and anti-thyroglobulin antibody (anti-Tg Ab) were performed in total thyroidectomy cases. An ultrasound of the cervical lymph nodes in all patients and the remaining lobes in patients with hemithyroidectomy was also performed.

#### 2.4.3. Yearly

An ultrasound of the cervical lymph nodes in all patients and the remaining lobes in patients with hemithyroidectomy was repeated. Biochemical analyses for serum TSH, FT4, Tg, and anti-Tg Ab were performed.

### 2.5. Outcome

Locoregional or distal recurrence of thyroid malignancy in the follow-up period and disease-free or overall survival of patients with I-PTM were the primary outcome parameters.

## 3. Results 

During the study period, total thyroidectomy and right and left hemithyroidectomy were performed in 249, 75, and 71 patients, respectively, for the treatment of benign surgical diseases of the thyroid gland among the total 395 patients. The females consisted of 75% of the patients. I-PTM was diagnosed on histopathological examination in 37 (9.4%) of the 395 patients. Moreover, 47% of our thyroid papillary cancer cases were PTM, of which 78.7% were incidentally discovered. I-PTM was diagnosed in 10.8% and 5% of female and male patients, respectively (Table 1).

The mean age of the 37 patients with I-PTM was 44.1 (16–71) years. More than half of the patients (56.8%) were aged between 41 and 60 years (Table 2).

The prevalence rates of I-PTM were 11.6% (29/249) in the total thyroidectomy specimens and 5.5% (8/146) in the unilateral hemithyroidectomy specimens (Table 3).

### 3.1. Pathology

Histopathological examination revealed 45 foci of PTM in 37 patients. The disease was unifocal in 30 and multifocal in 7 (18%) patients (6 cases of two and 1 case of three foci). Of the 45 foci, 25 (55.6%) were located in the left lobe (Table 4). Bilateral location of the PTM foci was observed in 6 (20.1%) of the 29 total thyroidectomy cases (Table 3).

The mean size of the PTM foci was 4.88 (1–10) mm, and 42.2%, 64.4%, and 71% of the foci were ≤3 mm, 5 mm, and 6 mm, respectively (Tables 5(a) and 5(b)).

Microscopic examination revealed thyroid capsule invasion in 2 (5.4%) of the 37 patients and in 2 (4.4%) of the 45 foci. These two foci measured 3 and 6 mm in two female patients aged 41 and 57 years, respectively. LVI was not reported in the PTM cases.

### 3.2. Adjuvant Treatment

Based on the clinical and ultrasound findings of the cervical lymph nodes and thyroid gland, we did not perform completion thyroidectomy in eight PTM cases with unilateral hemithyroidectomy. Patients with I-PTM did not receive postoperative RAI treatment. All patients received a suppressive dose of LT4 to achieve a serum TSH level of <0.25 uIU/mL.

### 3.3. Follow-Up

During the follow-up period of 102 (61–144) months, we did not find any negative changes in blood chemistry and ultrasound imaging. We also did not find any unfavourable locoregional or distant events as recurrence in the remaining thyroid lobes, metastasis in the cervical nodes, and distant metastasis.

## 4. Discussion

PTM is an indolent neoplasia, often asymptomatic and discovered incidentally [4]. Papillary microcarcinoma is accordingly found more frequently and often incidentally upon histopathological examination of surgical specimens from presumed benign thyroid disease [5]. Our study region was formerly a low iodine intake (endemic goitre) area where preventive iodine supplementation programme has been ongoing since the last 30 years. Several changes have been observed in medical and surgical thyroid diseases. The significant increase in the diagnostic rates of PTM, particularly the incidental findings after thyroid surgery, raises an important issue. We observed the diagnostic rate of I-PTM to be 78.7% among all the PTM cases, which corroborates our previous results. In our endemic region, the increased frequency of PTM and higher diagnostic rates of I-PTM indicate the occurrence of papillary cancer after iodine supplementation.

The presence of incidental thyroid cancer is an important issue in patients with benign thyroid diseases who underwent thyroid surgery. Postoperatively, we discovered that the thyroid gland harboured PTM in approximately 1 (9.4%) out 10 of our patients with benign thyroid disease. Previous studies have also reported the prevalence of I-PTM to be between 7.1% and 16.3% [6–10]. Based on the present study and previous results of prevalence, we suggest that the presence of incidental cancer is not an uncommon situation. During our study period in the endemic area, the proportion of PTM was 47% among all papillary thyroid cancer cases, and a vast majority (78.7%) of them showed incidental diagnosis, which indicates the importance of this pathology in the increasing rates of differentiated thyroid cancers. Lombardi et al. [11] reported the proportion of I-PTM to be 42%, of which 75.5% were incidental in an area with a high prevalence of goitre. Rosa Pelizzo et al. [3] have also reported an increase in the proportion of PTM from 35% to 56%, of which 60% were incidental. In another study, the proportion of PTM in papillary thyroid cancer cases was determined to be 49%, of which 58% were incidental [8]. The proportion of incidental cases among all PTM cases was found to be between 49% and 75.5% [8, 11–15]. Londero et al. [5] recently reported that age-standardised rates increased from 0.35 per 100,000 per year in 1996 to 0.74 per 100,000 per year in 2008. About 59% of PTM cases were identified incidentally, and a significant rise in incidence was found only for the incidental cases.

The increasing number of total thyroidectomies appears to be an important factor for the higher rate of incidental PTM. In our study, the incidence (11.6%) of I-PTM among total thyroidectomy cases was more than twice the incidence (5.5%) after hemithyroidectomy. Rosa Pelizzo et al. [3] reported an increase in the proportion of total thyroidectomies (from 67% to 78%) and PTM (from 35% to 56%). The prevalence of PTM is higher in patients with bilateral surgery [10].

The important features of papillary thyroid cancer include multifocality and bilateralism. Our incidence rate of 18% for the presence of two or more foci in the gland and the rate of 20% for the location of tumour foci in both lobes confirmed the pathological features of incidental PTM. Previous studies have also reported the multifocality rate of I-PTM to be between 13% and 44.1% [7–9, 15–19]. Some studies have reported multifocality as an independent risk factor for tumour recurrence [6, 8, 11, 14, 18, 20, 21]. On the other hand, the rate of recurrence is very low in patients with incidental PTM, which has been reported to range from 0% to 5% [6, 8, 11, 14, 15, 18, 19, 22]. Therefore, I-PTM cases with multifocality should be followed up more closely, despite the low risk of recurrence. Another pathological feature arising from our study was the rate of bilateralism (20%). Multiple foci were located in both lateral lobes in 20%–27.3% of patients with I-PTM [7, 9]. Based on the results of both the present and previous studies, the higher rate of bilateral tumour foci in both lobes draws our attention to the remaining lobes in patients with hemithyroidectomy. In such cases, sensitive imaging methods, such as ultrasound, should be used for the detection of nodules in the remaining lobes at both early postoperative and follow-up periods.

In general, incidental tumour foci are relatively smaller in size than nonincidental tumour foci. The average size of the tumour foci was <5 mm in patients with I-PTM [9, 15, 16, 18]. Our results confirmed the relatively small size (average 4.88 mm) of the tumour foci in the thyroid glands of patients with incidental PTM, of which 64% and 71% of foci were ≤5 mm and ≤6 mm, respectively. John et al. [15] reported that the tumour foci were ≤6 mm in 83% of such patients. Previous studies have reported tumour size as a risk factor for lymph node metastasis and recurrence. In general, tumour foci diameter >5 or 6 mm has appeared as an independent risk factor [6, 20, 21, 23, 24]. Therefore, patients with foci >6 mm in diameter should be followed up more closely. The recurrence rate of PTM is very low even among tumour foci > 6 mm, which has been reported to range from 0% to 5% [5, 6, 8, 14, 25–27].

Other important pathological features of thyroid malignancies are thyroid capsule invasion and LVI. The presence of LVI is a pathological feature showing the metastatic ability of malignancy. In our study, the absence of LVI in all our cases indicates the decreased ability of local and systemic spread of PTM. Previous studies have defined thyroid capsule invasion as extrathyroid or extracapsular spread, growth, extension, or invasion. Capsular invasion by PTM is an independent risk factor for tumour recurrence in the follow-up period [2, 6, 11, 12, 14, 20–23, 28]. Based on the rate of thyroid capsule invasion (5.4%) in the present study, we suggest that PTM with subcapsular localisation uncommonly invades the capsule of the thyroid gland. This rate has been reported to be 3.9% by John et al. [15] and 7.4% by Sakorafas et al. [9].

Locoregional and distant spread and recurrence of malignancy are the most important factors during the follow-up period. In our study, the absence of recurrence, lymph node, or distant metastasis indicates very low risk of tumour recurrence in patients with incidental PTM. The recurrence rate is 0% in some of the previous studies [12, 14, 19, 26]. In general, the overall recurrence rate was <2% in many case series [2, 5, 15, 22, 27]. Other studies have also reported a low recurrence rate between 2% and 5% [6, 8, 11, 13, 18, 25]. I-PTM is found in the thyroid tissue after surgical treatment of benign thyroid disease. Therefore, total thyroidectomy and unilateral hemithyroidectomy are usually performed in these cases. In this situation, is any adjuvant treatment required after the diagnosis of PTM? Completion thyroidectomy for hemithyroidectomy cases, RAI treatment, and suppression of TSH levels with LT4 treatment are the primary adjuvant procedures for patients with differentiated thyroid cancer. However, a very low rate of recurrence in a large series of I-PTM cases does not indicate the use of adjuvant treatment, except the suppression of TSH levels with LT4 treatment. After total excision of the gland, thyroidectomized patients receive L-thyroxin (LT4) replacement in order to maintain normal body function and TSH level in normal range [29]. On the other hand, well-differentiated malignant cells of papillary cancer have thyrotropin receptors in which the growth of differentiated malignant cells is controlled by TSH [30]. Therefore, inhibition of TSH secretion has beneficial effects on management of TSH-dependent tumour growth of well-differentiated malignancies that this secretion is inhibited by LT4 administration. Inhibition of TSH secretion should be provided by suppressive dose of LT4 in all patients with papillary cancer. The suppressive dose of LT4 is adjusted in individual basis by serum TSH level which is maintained between 0.1 and 0.25 uIU/mL according to patients' tolerability. In older patients, the dose of LT4 and suppression of TSH should be balanced with the cardiac risk [30]. After yearly follow-up the dose of LT4 can be decreased to maintain serum TSH levels between 0.5 and 1 uIU/mL in patients without unfavourable findings. In our study, surgical and nuclear adjuvant treatment was not attempted in any patients after a careful evaluation with imaging modalities and blood analysis. In our series of I-PTM cases, the absence of locoregional and distant recurrence during the follow-up period of 102 months confirmed the adequacy of the surgical treatment. Similarly, several previous studies have also reported no tumour recurrences after the surgical treatment of I-PTM [12, 14, 19, 26]. Based on our study and previous studies, we suggest that routine completion thyroidectomy after unilateral excision, lymph node dissection, and RAI application are unnecessary in most I-PTM cases.

In our endemic goitre area, diagnosis of I-PTM in thyroid tissue is not an uncommon situation after thyroid surgery for benign diseases. The prevalence of I-PTM increases parallel to the increase of total thyroidectomy rate for benign thyroid diseases. Multifocality and bilateralism are main pathologic features of PTM. Size of I-PTM foci in thyroid tissue is relatively small and the majority are smaller than 6 mm. Small foci of PTM create very low risk of lymph node metastasis and locoregional or distant recurrences in the follow-up period. The prognosis is excellent after surgical treatment and TSH suppression with LT4 administration. Routine adjuvant surgical and nuclear treatment as completion thyroidectomy, lymph node dissection, and RAI application is unnecessary in vast majority of patients due to low risk of recurrence. Such adjuvant procedures should be reserved for small number of recurrent cases discovered in the follow-up period.

## Figures and Tables

**Table 1:** Sex distribution of patients with surgical disease of the thyroid gland.

Patients	All	Female	Male
Thyroidectomy cases	395	295 (74.7%)	100 (25.3%)
Patients with I-PTM^*∗*^	37	32 (86.5%)	5 (13.5%)
Rate of I-PTM	9.4%	10.8%	5%

^*∗*^I-PTM: incidental papillary thyroid microcancer.

**Table 2:** Age distribution.

Age distribution	Patients with I-PTM^*∗*^
0–20 years	1 (2.7%)
21–40 years	13 (35.1%)
41–60 years	21 (56.8%)
61–80 years	2 (5.4%)

^*∗*^I-PTM: incidental papillary thyroid microcancer.

**Table 3:** Surgery of the thyroid gland and prevalence of I-PTM^*∗*^.

Surgery	Patients	Lobes		I-PTM	Foci	Bilateral
		Right	Left			
Total thyroidectomy	249 (63%)	249	249	29 (11.6%)	36	6 (20.1%)
Right hemithyroidectomy	75 (19%)	75	—	5 (6.7%)	6	—
Left hemithyroidectomy	71 (18%)	—	71	3 (4.2%)	3	—
Total	395	324	320	37 (9.4%)	45	—
		644				

^*∗*^I-PTM: incidental papillary thyroid microcancer.

**Table 4:** Location of I-PTM^*∗*^ foci in thyroid lobes.

Surgery location	Total thyroidectomy	Right hemithyroidectomy	Left hemithyroidectomy	Total
Right lobe	14	6	—	20 (44.4%)
Left lobe	22	—	3	25 (55.6%)
Total	36	6	3	45 (100%)

^*∗*^I-PTM: incidental papillary thyroid microcancer.

**(a):** 

Size	Right lobe	Left lobe	Total
0–5 mm	11	18	29 (64.4%)
6–10 mm	9	7	16 (35.6%)
Total	20	25	45

^*∗*^I-PTM: incidental papillary thyroid microcancer.

**(b):** 

Size	Right lobe	Left lobe	Total
0–3 mm	8	11	19 (42.2%)
4–6 mm	4	9	13 (28.9%)
7–10 mm	8	5	13 (28.9%)
Total	20	25	45

^*∗*^I-PTM: incidental papillary thyroid microcancer.
